# Authors’ Response to Peer Reviews of “Development and Assessment of a Point-of-Care Application (Genomic Medicine Guidance) for Heritable Thoracic Aortic Disease”

**DOI:** 10.2196/64436

**Published:** 2024-10-08

**Authors:** Rohan Patil, Fatima Ashraf, Samer Abu Dayeh, Siddharth K Prakash

**Affiliations:** 1 McGovern Medical School, University of Texas Health Science Center at Houston, Houston, TX, United States; 2 McWilliams School of Bioinformatics, University of Texas Health Science Center at Houston, Houston, TX, United States; 3 Department of Internal Medicine, John P and Kathrine G McGovern Medical School, University of Texas Health Science Center at Houston, Houston, TX, United States

**Keywords:** genomic medicine, point of care, thoracic aortic aneurysm, aortic dissection, decision support

*This is the authors’ response to the peer-review reports for “Development and Assessment of a Point-of-Care Application (Genomic Medicine Guidance) for Heritable Thoracic Aortic Disease.”*

## Round 1 Review

### Anonymous [1]

#### General Comments

*The Genomic Medicine Guidance (GMG) application described in this paper [2] needs to be made more intuitive for patients and clinicians to use. Additionally, genetic data integration needs to be expanded, clinical recommendations based on updated thoracic aortic aneurysms and dissections guidelines need to be updated frequently, and patient education materials need to be improved for clarity. It is also essential to make improvements for user feedback methods, multilingual support, accessibility, and strong data security. Enhancing the app’s effectiveness and usability requires a number of improvements, including customizable report options, better electronic health record integration, mobile device optimization, extensive training materials for clinicians, new research alerts, interactive tools like risk calculators, enhanced app performance, collaborative features, scalability to handle increased data loads, telehealth integration, support for custom user profiles, and community support features.*

#### Specific Comments

##### Major Comments

*1. How can the user interface be improved so that patients and physicians find it easier to use?*

Response: In lines 182‐185, we added “Long-term plans to improve the usability of GMG include a system to alert users when a similar variant is entered into the app, an optimized interface for mobile devices, multilingual support, and, in collaboration with the UTHealth Houston medical education team, enhancements for visually impaired users such as customizable colors and fonts, descriptive text, and screen readers.”

*2. How can the application include a wider variety of genetic data sources?*

Response: In lines 175‐179, we added “Future versions of GMG will leverage existing partnerships with cardiovascular specialists and the CardioGenomic Testing Alliance to incorporate gene-based care guidance for other adult-onset genetic cardiovascular diseases that are primarily managed by non-expert clinicians, such as hyperlipidemias, cardiomyopathies, and channelopathies. We designed a streamlined workflow to facilitate importation of clinical and genetic data into GMG by potential collaborators. Crowdsourcing through GMG will expand the clinical and genetic content over time.”

*3. How are clinical recommendations updated on a regular basis in accordance with the latest thoracic aortic aneurysms and dissections guidelines?*

Response: We changed one sentence and added one sentence to lines 126‐129 to clarify: “These recommendations are based on the 2022 ACC/AHA Guidelines for the Diagnosis and Management of Aortic Disease. As future guidelines are published, clinical recommendations in the app will be regularly updated to reflect new developments.”

*4. What improvements can be made to the application’s patient education resources to improve understanding?*

Response: In lines 187‐188, we added “We will collect demographic and survey data from patients to increase the relevance and clarity of GMG output for users with lower health literacy.”

*5. What changes are required to the application to increase its accessibility for users with disabilities?*

Response: In lines 183‐185, we added “...and, in collaboration with the UTHealth Houston medical education team, enhancements for visually impaired users such as customizable colors and fonts, descriptive text, and screen readers.”

*6. Is multilingual assistance for people who don’t speak English planned?*

Response: Yes. We added this statement to lines 182‐185 as above.

*7. How successful is the existing system for collecting user input, and how may it be improved?*

Response: In lines 179‐181, we added “We designed a streamlined workflow to facilitate importation of clinical and genetic data into GMG by potential collaborators.” In lines 188‐191, we added “We will expand and further automate data uploads into GMG as more users and clinical experts contribute data.”

*8. What more privacy and data security precautions are required?*

Response: To lines 104‐105, we added “All GMG data is stored on an encrypted server with terabytes of storage that is only accessible by members of the study team.” The clinical recommendations in GMG are based on publicly available clinical guidelines. Individual GMG users will be required to provide consent to be recontacted about their genetic results before they can exchange information.

*9. Does the application offer extra choices for customizing report generation?*

Response: To lines 129‐130, we added “GMG displays clinician and patient outputs that are optimized for mobile viewing and can be printed, downloaded, or emailed in PDF format.”

*10. What improvements may be made to the application’s integration with other electronic health record systems?*

Response: In lines 189‐191, we added “To increase access to GMG, we will also seek collaborations with Epic Systems Corporation and other healthcare software companies to integrate GMG content into electronic health records.”

*11. How can the performance of the application be enhanced on mobile devices?*

Response: In lines 182‐191, we added “Long-term plans to improve the usability of GMG include a system to alert users when a similar variant is entered into the app, an optimized interface for mobile devices,...”

*12. Are there enough resources available to teach practitioners who are not experienced with interpreting genetic data?*

Response: We modified the sentence in 118‐119 to read “For additional guidance, users may view sample test report forms from commercial genetic laboratories with highlighted variant information.”

*13. Could the application be coupled with an alert system for fresh study findings?*

Response: In lines 182‐183, we added “Long-term plans to improve the usability of GMG include a system to alert users when a similar variant is entered into the app...”

*14. What interactive features (risk calculators, for example) can be included in the application?*

Response: Currently, risk calculators are beyond the scope of GMG functions. Thank you for this advice. We will consider incorporating this feature into future versions of the application.

*15. How might the application’s general functionality and speed be enhanced?*

Response: The general functionality of GMG will be improved according to the plans that we outlined for future improvements in lines 182‐191. Some of these enhancements, particularly integration into the electronic health record, may also increase the speed of the application.

*16. What further elements may be included to promote cooperation between medical professionals and patients?*

Response: In lines 173‐181, we elaborated on the elements of GMG that promote collaboration: “GMG includes a modular and scalable genotype-phenotype database that can promote collaboration by connecting providers who enter similar genetic variants to resolve variants of uncertain significance or build case series to elucidate new disease phenotypes. Future versions of GMG will leverage existing partnerships with cardiovascular specialists and the CardioGenomic Testing Alliance to incorporate gene-based care guidance for other adult-onset genetic cardiovascular diseases that are primarily managed by non-expert clinicians, such as hyperlipidemias, cardiomyopathies, and channelopathies. We designed a streamlined workflow to facilitate importation of clinical and genetic data into GMG by potential collaborators. Crowdsourcing through GMG will expand the clinical and genetic content over time.”

*17. As the application grows, how will it handle higher user and data loads?*

Response: We added this sentence to lines 104 and 105: “All GMG data is stored on an encrypted server with terabytes of storage that is only accessible by members of the study team.”

*18. Is it feasible to incorporate telehealth functionalities for conducting distant consultations?*

Response: Currently this feature is not enabled in the application. Thank you for this advice. We will consider incorporating this feature into future versions of GMG.

*19. Is it possible for the application to accommodate unique user profiles for various user groups, such as patients, researchers, and doctors?*

Response: Currently this feature is not enabled in the application. Thank you for this advice. We will consider incorporating this feature into future versions of GMG.

*20. How can the application encourage the exchange of information and experiences through a community support feature?*

Response: We modified lines 173‐175 to add “GMG includes a modular and scalable genotype-phenotype database that can promote collaboration by connecting providers who enter similar genetic variants to resolve variants of uncertain significance or build case series to elucidate new disease phenotypes.” In lines 179‐181, we added: “We designed a streamlined workflow to facilitate importation of clinical and genetic data into GMG by potential collaborators. Crowdsourcing through GMG will expand the clinical and genetic content over time.”

### Anonymous [3]

#### General Comments

*This paper highlights an important application of a point-of-care application in guiding clinicians in their management of patients. It will be of interest to the community served by the journal.*

#### Specific Comments

##### Major Comments

*There needs to be some statistics on the “accuracy” of the application. For example, a number of expert clinicians in genomic medicine and its use (eg, 10) need to make clinical decisions without the use of the application, and then get 10 other clinicians who are not experts in genomic medicine to use the application and compare the level of agreement. A concordance study of this type needs to be done.*

Response: Thank you for your advice. We agree that this type of study is essential to demonstrate that the implementation of GMG can change clinical decision-making in a meaningful way. However, an implementation study is beyond the scope of the current manuscript.

### Reviewer MB [4]

#### General Comments

*This paper describes an application, GMG, a point-of-care tool to deliver concise clinical information about gene mutations that cause heritable cardiovascular diseases.*

#### Specific Comments

*This paper provides technical details about the application and its purpose, which is to collate data about genetic/genomic risks into a readily usable summary of clinical recommendations, particularly for nonexpert clinicians. This seems like a useful resource that could be updated and expanded as time goes on. I have some comments for the authors to address in order to strengthen the manuscript.*

##### Major Comments

*1. Section 3.6: Please add a little more information about the user reviews. Who were these application users? Did they have genetics training/expertise? When and how were they recruited to provide this feedback? Was their feedback acted on in any way? (These details may belong better in the Methods section.)*

Response: In lines 92‐96, we added a section on user feedback: “We implemented a Qualtrics survey for users to rate GMG in several categories, including usability, clarity, and educational content. The survey included free-response questions that invited users to discuss positive features and areas for improvement. From January to July 2023, the survey was sent to specialist clinicians, general cardiovascular clinicians, clinical geneticists, genetic counselors, and nurses.”

*2. Line 102: The text refers to Table 1, but I cannot find any table.*

Response: We added Table 1 to the main text as requested in line 112: “Table 1. The distribution of curated GMG variants by gene”

*3. Line 115: Producing “patient-friendly outputs” is much easier said than done. How did the developers ensure that outputs are actually patient friendly? Were patients involved in the project team and/or user-testing activities? Are outputs “friendly” for patients from diverse sociodemographic populations or only those with high literacy and education? Are any visual/graphical formats used in addition to text? After jotting down the queries above, I explored the website a bit myself. I would not consider the “patient friendly results” to be very friendly at all, especially for those with lower health literacy. It would be good to spell out in the manuscript exactly what steps have been taken in this direction so far and to acknowledge that there is more that could—and hopefully will—be done.*

Response: We deleted the term “patient-friendly” from lines 31 and 129. In lines 185‐188, we added “We acknowledge that the current version of GMG is not optimized for patients from diverse socio-demographic populations. We will collect demographic and survey data from patients to increase the relevance and clarity of GMG output for users with lower health literacy.”

*4. Line 167: Related to the above comments, the manuscript concludes with a throwaway line that the application “empowers patients to take an informed role in healthcare decisions.” Undoubtedly, the application developers aspire to “empower” patients, but the manuscript presents no evidence to back this up. If the authors have data on how the app affects patient-reported outcome measures, please add this into the manuscript. If not, please temper this concluding statement. Perhaps an evaluation of whether and how the application actually changes patient knowledge and participation in decision-making would be a valuable next step in the research agenda.*

Response: We changed lines 196‐197 as follows: “Additional studies are needed to evaluate how implementation of GMG can change clinician decision-making and increase patient insight into heritable cardiovascular diseases.”

##### Minor Comments

*5. Line 42‐43: Please provide brief examples of “timely individualized interventions that can prevent deaths.”*

Response: In line 42, we added “such as titration of medical therapies or preventative surgical repair of the aorta.”

*6. Line 44: Define “ACC/AHA.*

Response: We expanded the acronyms “ACC” and “AHA” in lines 44 and 45 as requested.

*7. Line 105: Define “HTAD.*

Response: We replaced “HTAD” with “TAD,” which we defined earlier in the text.

### Reviewer MT [5]

#### General Comments

*This paper describes the development and implementation of a novel application designed to assist clinicians and patients in managing heritable thoracic aortic diseases (HTADs) through accessible genomic information. The GMG application, developed using REDCap, integrates genetic data with clinical recommendations to provide comprehensive guidance on diagnosis, treatment, and surveillance of HTAD. Preliminary user feedback indicates high usability and positive impact on clinical guidance, suggesting the GMG application could significantly contribute to personalized patient care and potentially influence clinical practices toward better management of HTAD.*

*This manuscript is well structured, presenting a clear problem statement, detailed development methodology, results from initial user feedback, and a discussion on the implications of the application in clinical settings. It addresses a critical gap in the application of genomic medicine in clinical practice, particularly in the management of heritable aortic diseases. Overall, the manuscript presents a strong case for further research and development.*

#### Specific Comments

##### Major Comments

*1. The article would benefit from a more detailed analysis of user feedback, including data on the application’s impact on clinical decision-making and patient outcomes.*

Response: In lines 92‐96, we added a section to describe the method of collecting user feedback: “We implemented a Qualtrics survey for users to rate GMG in several categories, including usability, clarity, and educational content. The survey included free-response questions that invited users to discuss positive features and areas for improvement. From January to July 2023, the survey was sent to specialist clinicians, general cardiovascular clinicians, clinical geneticists, genetic counselors, and nurses.”

Thank you for your advice. We agree that a study to demonstrate that implementation of GMG can change clinical decision-making and patient outcomes is essential. However, an implementation study is beyond the scope of the current manuscript.

*2. Consider including possibilities for future updates and challenges in a broader implementation.*

Response: We included a paragraph detailing planned future updates to GMG in lines 182‐191. We updated the Conclusion to add: “Additional studies are needed to evaluate how implementation of GMG can change clinician decision-making and increase patient insight into heritable cardiovascular diseases.”

## Round 2 Review

### Anonymous [3]

#### General Comments

*With respect to my initial recommendation: There needs to be some statistics on the “accuracy” of the application. For example, a number of expert clinicians in genomic medicine and its use (eg, 10) need to make clinical decisions without the use of the application, and then get 10 other clinicians who are not experts in genomic medicine to use the application and compare the level of agreement. A concordance study of this type needs to be done.*

#### Specific Comments

##### Major Comments

*This should not be beyond the scope of the manuscript. Without this information, the manuscript is not high impact but low impact or a niche interest. I recommend you pursuing this recommendation.*

Response: As requested, we created a user survey to determine the efficacy of the application to guide clinical management decisions. Users without formal genetic training were provided with a sample genetic test report and a set of questions that they were directed to answer using the GMG output. We created two new sections of the manuscript, 1.2.4 and 1.3.7, to describe the efficacy test and results. Most clinician users were able to make correct recommendations based on GMG data. We will expand these surveys to include more users as the application is rolled out to new clinics.

## Abbreviations

GMG: Genomic Medicine Guidance
HTAD: heritable thoracic aortic disease
